# Editorial: Plant biodiversity and genetic resources: their utilization for crop improvement

**DOI:** 10.3389/fgene.2024.1513972

**Published:** 2024-11-13

**Authors:** Fakhriddin N. Kushanov, Shoupu He, Jinesh Patel

**Affiliations:** ^1^ Institute of Genetics and Plant Experimental Biology, Academy of Sciences of the Republic of Uzbekistan, Tashkent, Uzbekistan; ^2^ Department of Genetics, National University of Uzbekistan, Tashkent, Uzbekistan; ^3^ Department of Biology, Namangan State University, Namangan, Uzbekistan; ^4^ State Key Laboratory of Bio-breeding and Integrated Utilization, Institute of Cotton Research Chinese Academy of Agricultural Sciences, Anyang, China; ^5^ Crop, Soil, and Environmental Sciences, Auburn University Auburn, Auburn, AL, United States

**Keywords:** plant biodiversity, genetic resources, crop improvement, genomics, stress tolerance, 11 underutilized crops, wild relatives, molecular breeding 12

## Introduction

As global agriculture faces unprecedented challenges—from climate change to food insecurity—plant biodiversity and genetic resources have emerged as crucial assets for crop improvement. Harnessing the genetic diversity of both cultivated plants and their wild relatives provides opportunities to develop resilient crops and crop varieties capable of withstanding environmental stresses, thereby improving yields and ensuring yield stability. Depending on parental selection, the nutritional content of crop varieties may also be improved. Recent advances in genomic tools, such as high-throughput sequencing and bioinformatics, have enabled researchers to explore and utilize these genetic resources more effectively. This Research Topic, “Plant Biodiversity and Genetic Resources: Their Utilization for Crop Improvement,” highlights the latest innovations in the field and showcases how genomic tools are being applied to improve crop performance and sustainability of production.

## Genomic insights into crop improvement: a comprehensive review

Plant genomics has facilitated significant progress in understanding the genetic basis of key traits in agricultural crops. In turnip rape (*Brassica rapa*), genomic prediction techniques have been successfully applied to accelerate breeding for traits such as days to flowering, seed yield, and pod shattering resistance. By analyzing the genomic diversity of 170 turnip rape accessions, Alemu et al. identified single nucleotide polymorphisms (SNPs) linked to various agro-morphological traits, providing breeders with valuable information on improving crop resilience and productivity.

Soybean (*Glycine max*), a major oilseed crop, is facing challenges from diseases such as Cercospora leaf blight (CLB). Patel et al. employed genome-wide association studies (GWAS) to identify SNPs associated with CLB resistance, highlighting key genomic regions that could be leveraged to develop disease-resistant soybean varieties.

Flax (*Linum usitatissimum*), a dual-purpose crop valued for both its oil and fiber, has also benefited from genomic advances. Dvorianinova et al. presented the first high-quality genome sequence of a dehiscent flax variety, shedding light on the genetic factors controlling traits like capsule dehiscence and fiber production. These insights have significant implications for flax breeding programs aimed at improving both yield and quality (Dvorianinova et al.). Similarly, in radish (*Raphanus sativus*), Park et al. developed a chromosome-level genome assembly and identified genes related to agronomically important traits, such as stress resistance, enabling the development of more robust radish cultivars.

Underutilized pulses, often grown in marginal environments, hold great potential for improving food security and resilience. Dwivedi et al. explored the genetic and genomic resources of 13 underutilized legume species, highlighting their potential for breeding climate-resilient crops. Advances in genome sequencing have made it possible to identify key genes associated with traits like drought tolerance and nutritional content, paving the way for the wider adoption of these species in modern agriculture (Dwivedi et al.).

### Harnessing genetic diversity from wild relatives

RNA-seq studies in genetically diverse individuals have gained significant attention due to their ability to rapidly identify the genetic architecture of a trait of interest. He et al. conducted a comparative transcriptomic analysis on fiber samples collected at different developmental stages of two upland cotton (*Gossypium hirsutum*) accessions with contrasting fiber qualities. By identifying differentially expressed genes (DEGs) involved in fiber development, their study provides a molecular framework for improving cotton fiber quality through breeding programs (He et al.).

Wild relatives of the *Moringaceae* family offer valuable genetic resources for improving stress tolerance and nutritional content in domesticated crops. AbdAlla et al. sequenced the complete chloroplast of *Moringa peregrina*, an endangered species native to Egypt, revealing its phylogenetic relationships with other members of the Brassicales order. This genomic information is critical for conservation efforts, understanding the evolutionary history of *M. peregrina* in complex Brassicales order families, and for potential use in breeding programs aimed at enhancing abiotic stress resistance (AbdAlla et al.).

Capsicum species, including chili pepper (*Capsicum annuum*), have undergone significant domestication, leading to a reduction in genetic diversity for certain traits. Lopez-Moreno et al. performed QTL mapping to identify genomic regions associated with 19 domestication and agronomic traits such as fruit size, seedlessness, and growth habit. Their findings underscore the importance of utilizing wild relatives like *C. annuum var. glabriusculum* to reintroduce genetic diversity into breeding programs, thereby improving traits that have been lost during domestication (Lopez-Moreno et al.).

### Applications of genomics in fiber and oil crops

The application of genomics to fiber and oil crops such as cotton and flax continues to yield promising results. In upland cotton, the identification of key genes involved in fiber elongation and secondary cell wall biosynthesis has enabled researchers to develop molecular markers for improving fiber quality and yield (He et al.). Meanwhile, in flax, genome sequencing has provided valuable insights into the genetic regulation of both fiber production and seed oil content, paving the way for the development of superior flax varieties (Dvorianinova et al.).

### The potential of underutilized species and landraces

Underutilized legume species and landraces represent an important resource for diversifying agriculture and improving resilience to climate change. Dwivedi et al. emphasized the potential of crops like African yam bean, winged bean, and horse Gram to improve food security in regions affected by environmental stress. Genomic research has revealed key genes responsible for traits such as drought tolerance, nitrogen fixation, and high protein content, making these crops valuable candidates for future breeding programs (Dwivedi et al.).

## Conclusion

The research presented in this collection underscores the critical role of plant biodiversity and genetic resources in addressing the global challenges facing agriculture today. Genomic tools have opened up new possibilities for exploring and utilizing the genetic diversity found in both cultivated crops and their wild relatives. By integrating these resources into breeding programs, we can develop crops that are not only more productive but also more resilient to the environmental stresses of the 21st century. As the field of genomics continues to advance, the potential for harnessing plant biodiversity for crop improvement will only grow, offering new solutions for ensuring global food security and sustainability of crop production.

